# Serum lipids might improve prostate-specific antigen sensitivity in patients undergoing transrectal ultrasonography-guided biopsy for suspected prostate cancer: A pilot study

**DOI:** 10.1080/2090598X.2019.1626126

**Published:** 2019-06-12

**Authors:** Ahmed M. Harraz, Nora Atia, Amani Ismail, Hassan Abol-Enein, A.F. Abdel-Aziz

**Affiliations:** a Department of Urology, Urology and Nephrology Center, Mansoura University, Mansoura, Egypt; b Department Biochemistry, Mansoura University, Mansoura, Egypt; c Department of Clinical Pathology, Urology and Nephrology Center, Mansoura University, Mansoura, Egypt

**Keywords:** Prostate cancer, PSA, metabolic syndrome, TRUS-guided biopsy, serum lipids, LDL

## Abstract

**Objectives**: To investigate the potential use of body mass index (BMI) and serum lipids in improving prostate-specific antigen (PSA) sensitivity in patients undergoing biopsy for suspicion of prostate cancer, as there is an established relationship between metabolic syndrome, obesity and serum lipids with prostate cancer.

**Patients and methods**: A pilot study was conducted in a tertiary referral centre between June 2016 and August 2017 of patients undergoing transrectal ultrasonography (TRUS)-guided biopsy. After the result of TRUS-biopsy, the first 50 patients diagnosed with prostate cancer (study group) and those with no prostate cancer (control group) were enrolled. BMI, serum PSA level, fasting blood sugar and lipid profile (e.g. cholesterol, triglycerides, low-density lipoprotein [LDL] and high-density lipoprotein [HDL]), were compared between the groups.

**Results**: Higher BMI, cholesterol, LDL and lower HDL together with PSA were significantly associated with a positive biopsy. On multivariate analysis, LDL (odds ratio [OR] 5.3, 95% confidence interval [CI] 1.2–24.9; *P* = 0.03) and total PSA level (OR 12.9, 95% CI 4.7–35; *P* < 0.001) were independent predictors of a positive biopsy. A combination of LDL <80 mg/dL and PSA level <26 ng/mL threshold values determined by receiver operating characteristic curve analysis, had a sensitivity and specificity of 94% and 28%, respectively; whilst, the negative (NPV) and positive predictive values were 82.4% and 56.6%, respectively. The sensitivity and NPV of the combination was significantly higher than that of PSA level alone (94% vs 72% and 82.4% vs 75%, respectively; *P* < 0.001).

**Conclusions**: Serum lipids might have a role in the diagnosis of prostate cancer and could be used as an adjunct to PSA measurement to improve sensitivity and avoid unnecessary biopsies.

**Abbreviations:** AUC: area under the curve; BMI: body mass index; FBS: fasting blood sugar; HDL: high-density lipoprotein; LDL: low-density lipoprotein; LOX-1: lectin-like oxidised LDL receptor-1; OR: odds ratio; ROC: receiver operating characteristic; RP: radical prostatectomy; TG: triglyceride

## Introduction

Metabolic syndrome is an array of risk factors that are closely related to the development of obesity, diabetes mellitus, and cardiovascular disease. The underlying pathophysiological mechanisms are complex including, but not limited to, visceral adiposity, atherogenic dyslipidaemia, and endothelial dysfunction [1]. These factors combined together have a possible causal relationship in the development of various diseases including prostate cancer [2,3]. It has been postulated that free fatty acids, adipokines secreted from fat cells, aromatase activity, insulin resistance and IGF-1 are contributing factors [2].

A handful of studies have shown a close link between obesity, serum lipids and prostate cancer [3,4]. Elevated triglycerides (TGs) and low high-density lipoprotein (HDL) levels were significantly associated with high-grade prostate cancer, albeit this relationship was not consistent between reports [5–8]. Moreover, low HDL was associated with adverse pathological findings after radical prostatectomy (RP) [9]. Likewise, body habitus has a proven role in the pathogenesis of prostate cancer [10,11].

In lieu of the potential implications of obesity and its related factors on the development of prostate cancer, we investigated in a prospective pilot study whether body mass index (BMI) and serum lipid profile might have a role in optimising prostate cancer diagnosis when combined with serum PSA level.

## Patients and methods

A pilot study was conducted in a tertiary referral centre, between June 2016 and August 2017, of patients undergoing TRUS-guided biopsy for suspicion of prostate cancer. Patients were referred for biopsy if they had an abnormal DRE and or a serum PSA level of >4 ng/mL; and after the exclusion of potential non-cancerous causes of elevated PSA, e.g. infections, retention, and instrumentation. The study was approved by the local Institutional Review Board and Medical Ethics Committee.

Patients referred for TRUS-guided biopsy were notified of the study and those who agreed to participate did so via a written consent. After TRUS-guided biopsy, the first 50 patients diagnosed with prostate cancer and those with no prostate cancer were enrolled as the study and control groups, respectively. In cases of raised clinical suspicion with negative biopsies, repeat biopsies were taken to confirm the absence of cancer. Patients were asked to fast for 16 h before withdrawing a blood sample; BMI was calculated at the same time as blood sample collection. Retrieved data included a brief medical history, with an emphasis on the current medications of the patient. Blood sample assessments included: serum PSA (ng/mL); fasting blood sugar (FBS); and lipid profile, e.g. cholesterol, TGs, low-density lipoprotein (LDL), and high-density lipoprotein (HDL).

The biopsy technique was standardised and performed by a dedicated interventional radiologist. Patients were asked to do a cleansing enema at home on the day of the biopsy. Then, in lithotomy position, TRUS-guided biopsies were taken using BK-ultrasound™ imaging systems (BK Medical, Mileparken, Denmark); 12 cores were routinely taken unless otherwise indicated, with less cores due to limited patient’s tolerability or extra cores for suspicious lesions.

### Study outcome and statistical analysis

The primary outcome of the study was to investigate whether BMI and serum lipid profile would improve the sensitivity of PSA, and consequently avoid unnecessary biopsies. All variables were described as mean and SD apart from the total PSA level that was described as median and range, being non-parametrically distributed. For comparisons between means, the Student’s *t*-test was used, whilst the Mann–Whitney *U*-test was used for the differences between medians. Receiver operating characteristic (ROC) curve analysis was used to define threshold values associated with the presence of prostate cancer at biopsy and consequently transforming scale to dichotomous variables.

The *P* value for the frequencies in each category was determined using the chi-squared or Fisher’s exact test. Univariate and multivariate binary logistic regression models were used for determining independent factors associated with prostate cancer presence at biopsy. From the independent predictors, a combination of results was tested against PSA level alone as an indicator for prostate biopsy. Sensitivity analysis was done using the McNemar test. All statistical analyses were performed using the IBM Statistical Package for the Social Sciences (SPSS®) version 25 (SPSS Inc., IBM Corp., Armonk, NY, USA) and MedCalc® version 18.9.1 (MedCalc, Ostend, Belgium), and a *P *< 0.05 was considered to indicate statistical significance.

## Results

### Demographics

The mean (SD) age of the study population was 66.7 (8.6) years and the BMI was 29.6 (5.5) kg/m^2^. There was no significant difference in the presence of hypertension [12 (24%) and 13 (26%), *P* = 0.8] or diabetes mellitus [13 (26%) and 9 (18%): *P* = 0.3] in the prostate cancer and control groups, respectively. None of the patients received antihyperlipidaemic medications. DRE was suspicious in 18 (36%) patients diagnosed with prostate cancer vs 11 (22%) with negative biopsy (*P* = 0.1). The median (range) total prostate volume was 70 (20–268) mL and 90 (30–344) mL for the prostate cancer and control groups (*P* = 0.001), while the volume of the transitional zone was 37 (20–211) mL and 59 (20–240) mL (*P* < 0.001), respectively. The TRUS-guided biopsy results revealed prostatic adenocarcinoma with Gleason Score 6, 7, 8, 9, in eight (16%), 24 (48%), 14 (28%) and four (8%) patients, respectively.

### ROC curve analysis

**Figure 1.**
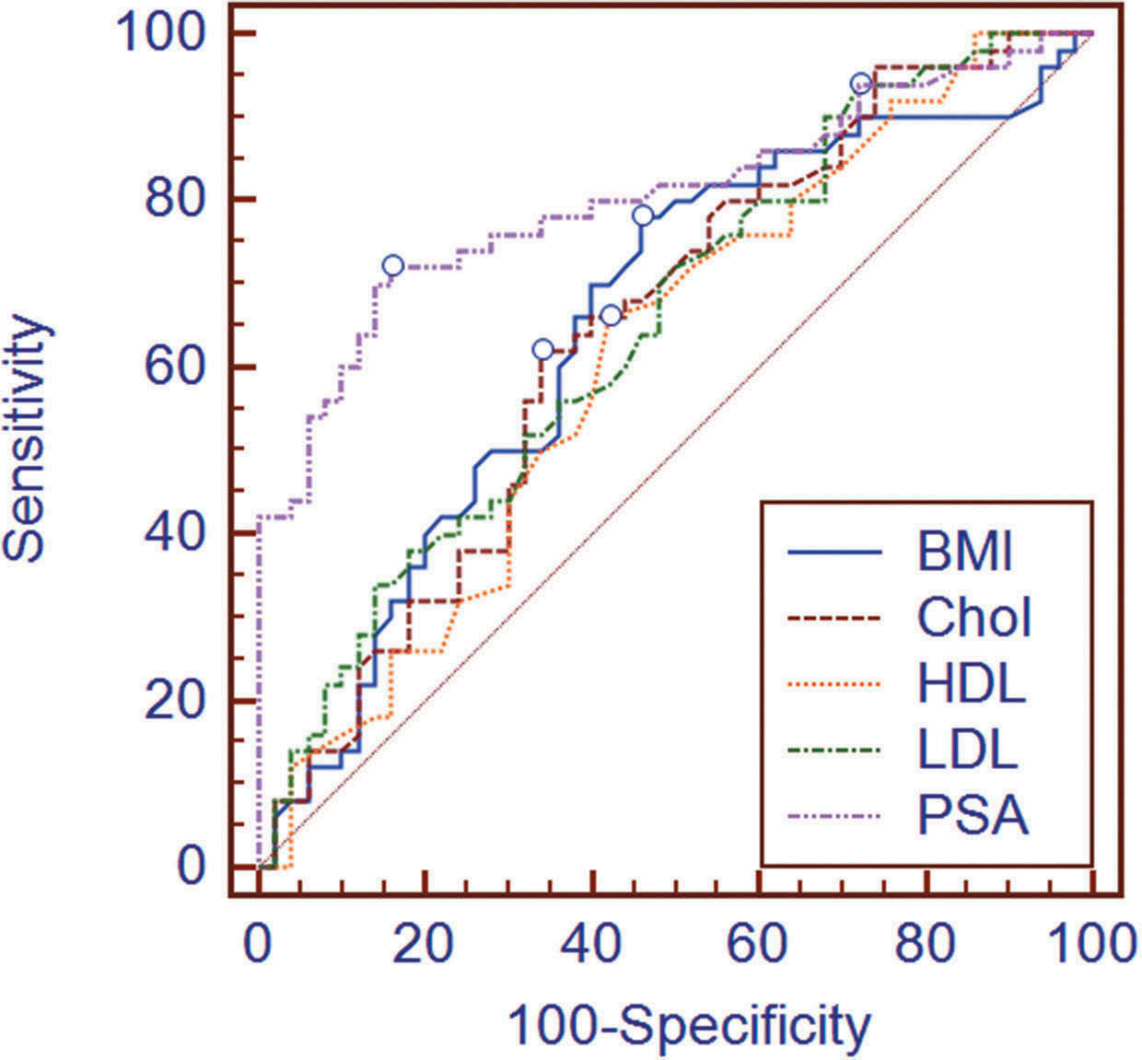
ROC curve analysis of BMI and serum lipid profiles as a diagnostic tool for the presence of prostate cancer on biopsy. Chol: cholesterol.

The threshold points associated with a positive biopsy were ≥27.5 kg/m^2^ for BMI (area under the curve [AUC] 0.65; *P* = 0.007; sensitivity 78%; specificity 54%), ≥200 mg/dL for cholesterol (AUC 0.64; *P* = 0.009; sensitivity 62%; specificity 66%), <40 mg/dL for HDL (AUC 0.61; sensitivity 66%; specificity 58%), ≥80 mg/dL for LDL (AUC 0.65; *P* = 0.006; sensitivity 94%; specificity 28%), and ≥26 ng/mL for total PSA level (AUC 0.8; sensitivity 72%; specificity 84%). On the contrary, no values were calculated for age, FBS or TGs being non-significantly associated with prostate cancer at biopsy. Figure 1 shows the results of the ROC curve analysis.

### Univariate and multivariate analysis

When analysed as scale and dichotomous variables, higher BMI, cholesterol, LDL and lower HDL, together with PSA level were significantly associated with a positive biopsy (Table 1). On multivariate analysis, LDL (odds ratio [OR]: 5.3; 95% CI 1.2–24.9; *P* = 0.03) and total PSA level (OR 12.9; 95% CI 4.7–35; *P* < 0.001) were independent predictors of a positive biopsy.

**Table 1.** The difference in values of serum lipid parameters and PSA amongst patients diagnosed with and without prostate cancer at biopsy.

Variable	Control	Prostate cancer	*P*
FBS, mg/dL, mean (SD)	108.7 (33.8)	101.7 (19)	0.2
TG, mg/dL, mean (SD)	138.3 (77)	138.2 (52)	0.9
BMI, kg/m^2^			
Scale, mean (SD)	28.5 (5.4)	30.8 (5.4)	0.03
Dichotomous, *n* (%)			
<27.5	27 (71.1)	11 (28.9)	0.001
≥27.5	23 (37.1)	39 (62.9)	
Cholesterol, mg/dL			
Scale, mean (SD)	187.9 (41.3)	208 (36.2)	0.01
Dichotomous, *n* (%)			
<200	33 (63.5)	19 (36.5)	0.005
≥200	17 (35.4)	31 (64.6)	
HDL, mg/dL			
Scale, mean (SD)	41.7 (11.3)	37.3 (8)	0.02
Dichotomous, *n* (%)			
<40	29 (63)	17 (37)	0.01
≥40	21 (38.9)	33 (61.1)	
LDL, mg/dL			
Scale, mean (SD)	106.5 (39.2)	127.7 (37.5)	0.007
Dichotomous, *n* (%)			
<80	14 (82.4)	3 (17.6)	0.003
≥80	36 (43.4)	47 (56.6)	
PSA level, ng/mL			
Scale, median (range)	13.8 (6–74)	57.7 (5.4–119)	<0.001
Dichotomous, *n* (%)			
<26	42 (75)	14 (25)	<0.001
≥26	8 (18.2)	36 (81.8)	

### Sensitivity analysis

Based on the results of the regression model, a combination of LDL <80 mg/dL and PSA level <26 ng/mL were considered as a new test in which a negative test result was considered if both variables were negative and a positive test result if both variables were positive or discordant. The sensitivity of the new test was 94% and the specificity 28%, whilst the positive predictive value and negative predictive value (NPV) were 56.6% and 82.4%, respectively. Comparison between the sensitivity and NPV of the new test and those of total PSA level alone showed improved sensitivity from 72% to 94% and the NPV from 75% to 82.4% (*P* < 0.001).

## Discussion

The results of the present pilot study confirm the close association between serum lipids and prostate cancer risk in patients with elevated PSA levels. Furthermore, serum LDL could be used to significantly improve the sensitivity and the NPV of PSA in patients undergoing TRUS-guided biopsy.

The relationship between serum lipids and prostate cancer risk and prognosis has been explored both on the genetic and clinical levels. Andreassen et al. [12] found polygenic overlap between serum lipid parameters and prostate cancer predisposition, and identified 17 pleiotropic gene loci between prostate cancer and LDL, and prostate cancer and TGs, although this was not demonstrated on a Mendelian randomisation analysis [13]. In a retrospective analysis of 3102 Chinese patients, a high level of serum cholesterol and LDL and lower level of HDL were significantly associated with patients diagnosed with prostate cancer, which is in concordance with the present study [14]. Likewise, hypertriglyceridaemia showed a weak positive but non-significant association with prostate cancer. On the other hand, HDL has been shown also to significantly correlate with high-grade prostate cancer at diagnosis, as shown in the *post hoc* analysis of the REduction by DUtasteride of prostate Cancer Events (REDUCE) study [15].

Besides a potential role in prostate cancer diagnosis, a serum lipogram has been shown to have prognostic value post-RP. Zhang et al. [16] analysed the association between preoperative serum lipids on post-RP histopathological features. They found an independent predictive relationship between higher cholesterol and lymph node invasion, and higher LDL and Gleason score. In a retrospective review of 843 statin-naïve RP patients, hypertriglyceridaemia was a significant predictor of prostate cancer recurrence, moreover, amongst men with dyslipidaemia, there was a 9% increased and 39% decreased risk of recurrence with each 10 mg/dL increase in cholesterol and HDL, respectively [17].

In the present study, LDL was the only parameter that demonstrated independent prediction of a positive biopsy. LDL excess has been shown to heighten the risk of cancer development in different reports. Furuya et al. [18] reported that LDL receptors, upregulated with the use of statins, played a crucial role in prostate cancer cell growth. In addition, lectin-like oxidised LDL receptor-1 (LOX-1) is highly expressed in vascular organs and is a receptor for oxidised LDL [19]. Once activated, several tumorigenic processes ensue including the expression of adhesion molecules, pro-inflammatory signalling pathways and proangiogenic proteins, including nuclear factor κ-light-chain-enhancer of activated B cells (NF-κB) and vascular endothelial growth factor (VEGF). Gonzalez-Chavarria et al. [20] have shown that LOX-1 expression is mandatory for tumour growth in nude mice. Furthermore, receptor activation promotes actin cytoskeleton restructuring and raising prostate cancer cell invasion.

In contrast to our present study, Wettstein et al. [21] reported that a lower level of LDL was an independent predictor of prostate cancer recurrence in patients undergoing RP. The possible explanation was that prostate cancer is dependent on cholesterol for metabolism and the lower levels of LDL might reflect the highly aggressive features of the tumour. Nevertheless, they were not able to demonstrate whether a lower LDL level was a modifiable risk factor or a result of increased tumour metabolism.

We observed a higher threshold value for PSA level that was significantly associated with prostate cancer detection compared with previously published reports. In a recent study, Leal et al. [22] identified the sensitivity of PSA levels of 3 and 4 ng/mL stratified by patient age in the detection of biopsy confirmed prostate cancer. On the other hand, others have used threshold values for PSA-density for improving prostate cancer detection at biopsy [23]. It is to be noted that these threshold values were determined based on screening programmes, which is not the same situation in the present study where all patients were referred for TRUS and biopsy, and consequently, had higher PSA levels. In addition, it is imperative to consider that ROC curve analysis determines the point of maximum sensitivity and specificity rather than detecting the lowest sensitivity at the expense of specificity. Therefore, higher threshold values are obtained.

The present study is the first to clinically implicate the association between serum lipids as a potential biomarker for enhancing prostate cancer detection at biopsy. We identified a threshold value that could be used to improve the sensitivity and NPV of PSA, and consequently avoiding unnecessary biopsies. Nevertheless, the present study is a pilot study that needs to be designed on a larger scale. Secondly, subject enrolment did not follow a random pattern, as the first 50 patients from each group were included, and consequently has the probability of selection bias. In addition, multiparametric MRI, although to a lesser extent would be of value in patients with such high PSA levels, was not utilised in this cohort as it is not routinely practiced in our institution. Lastly, sensitivity analysis was compared in relation to a total PSA threshold determined based on a small sample of population. Hence, the present results require validation in a larger cohort.

## Conclusions

Serum lipids were significantly associated with the diagnosis of prostate cancer. LDL could be used as an adjunct to PSA for patient stratification and improving the sensitivity and NPV for TRUS-guided biopsy result, and consequently to possibly avoid unnecessary biopsies. Implementing serum lipids as a potential biomarker in future studies is encouraged to further elucidate its role.
